# Access to comprehensive emergency obstetric and newborn care facilities in three rural districts of Sindh province, Pakistan

**DOI:** 10.1186/s12961-015-0042-7

**Published:** 2015-11-25

**Authors:** Muhammad Shahid Ansari, Rabia Manzoor, Nasim Siddiqui, Ahsan Maqbool Ahmed

**Affiliations:** Provincial Health Development Centre (PHDC) Sindh, Jamshoro, Pakistan; Development Strategies, Islamabad, Pakistan

**Keywords:** Access, Affordability, EmONC, C-EmONC facility, Public sector facility, Private sector facility, Secondary delay, Travel cost, Travel time

## Abstract

**Background:**

Pakistan’s maternal and child health indicators remain unacceptably high, with a maternal mortality ratio of 276 per 100,000 live births and a neonatal mortality rate of 55 per 1,000 live births. Provision of basic and comprehensive emergency obstetric and newborn care is mandated by the government; however, coverage, access, and utilisation levels remain unsatisfactory, with the situation in Sindh province being amongst the worst in the country. This study attempted to assess access to comprehensive emergency obstetric and newborn care (C-EmONC) facilities and barriers hampering access in Sindh.

**Methods:**

One public sector hospital in each of three districts in Sindh province providing C-EmONC services were selected for a facility exit survey. A cross-sectional household survey and focus group discussions were conducted in the catchment population of these hospitals.

**Results:**

Overall, 82% and 96% of those who utilised a public or private C-EmONC facility, respectively, incurred out-of-pocket expenditure. As expected, those living more than 5 km from the facility reported higher mean expenditure than those living within 5 km of the facility. More than half of the respondents (55%) among public sector users and the majority (71%) of private sector users could not afford travel costs. More than one third (35%) of public sector users and about two thirds (64%) of private sector users who could not afford travel costs took loans. The proportion of respondents who took loans was higher among those living more than 5 km of the health facility compared to those living within a 5 km distance. The majority of respondents (70%) in the community survey chose to go to a private sector C-EmONC facility. In addition to poverty, in terms of sociocultural access, religious and ethnic discrimination and the poor attitude of facility staff were amongst the most important barriers to accessing a C-EmONC facility.

**Conclusions:**

C-EmONC facilities in both the public and private sectors may simply not be accessible and affordable for the vast majority of poor and marginalised women in targeted districts.

## Background

Improving maternal, newborn, and child health indicators remains one of the most important global health challenges, especially in developing countries like Pakistan, where the situation remains unsatisfactory. The Government of Pakistan, with support from a number of United Nations organisations and development partners, have undertaken a range of initiatives aimed at improving maternal, newborn, and child health [1]. Despite these efforts, Pakistan’s maternal and child health indicators remain unacceptably high with a maternal mortality ratio of 276 per 100,000 live births [2] and a neonatal mortality rate of 55 per 1,000 live births [3]. The country is lagging behind in achieving Millennium Development Goal targets of maternal mortality for 2015, though WHO estimates indicate that Pakistan is making progress [4].

Basic and comprehensive emergency obstetric and newborn care (B- and C-EmONC) services are provided at various levels of the governmental health system. Primary healthcare facilities provide B-EmONC, including the provision of uncomplicated delivery care, basic resuscitation, and care for newborns. Secondary level facilities provide C-EmONC, including the provision of complicated delivery care and caesarean deliveries as well as advanced resuscitation and care for newborns. In the study site in Sindh, the health system consists of basic health units, rural health centres, Taluka hospitals, and district civil hospitals. Private sector facilities providing C-EmONC services are mostly present within the catchment area of public sector secondary care facilities. While basic health units and rural health centres act as primary care facilities providing B-EmONC services, Taluka hospitals and district civil hospitals provide 24-hour C-EmONC services. However, coverage, access, and utilisation levels remain unsatisfactory [5]. A previous situation analysis of public sector emergency obstetric care facilities in Sindh province depicted stark deficiencies in service availability, distance, transport accessibility to reach facilities, and capacity of facilities to extend requisite 24-hour C-EmONC services at the secondary level: 12% of facilities did not provide services after 2:00 p.m. and 24-hour coverage for complicated deliveries was provided in only 88% of secondary care hospitals. Transport for referrals was available in only 35% of the facilities and only 33% were equipped to offer obstetric care [6].

More than a decade after the last situation analysis, the Provincial Health Development Centre of the Government of Sindh conducted two surveys and focus group discussions (FGDs) to explore accessibility and factors responsible for inequities in and barriers to access to C-EmONC facilities, both in public and private sectors. This paper presents results from all three studies examining access to C-EmONC facilities and provides future directions for policy uptake to improve access to C-EmONC.

## Methods

### Study setting

The study was conducted in three districts of Sindh province, namely Ghotki, Jamshoro, and Tharparkar, representing northern, central, and southern parts of Sindh and chosen because they had a higher proportion of the population with lower economic profiles. In each district, one public sector hospital providing C-EmONC services was purposefully selected to conduct a facility exit survey. A cross-sectional community survey and FGDs were conducted in the catchment population of the selected hospitals to complement the exit survey. All three linked studies (i.e. facility exit survey, community survey, and FGDs) were conducted in the three districts from January to June 2013.

### Facility exit survey

#### Subject selection

Women who experienced common obstetric problems, such as antepartum or postpartum haemorrhage, complications from abortion, prolonged or obstructed labour, eclampsia/pre-eclampsia, or sepsis, and neonates who had any illness at the time of interview were eligible for inclusion in the exit survey. Female doctors and/or gynaecologists who treated the patients recorded their history, diagnosis, and treatment on the outpatient department register, an instrument of the district health information system. Every third patient who met the eligibility criteria on the day of survey was selected from the register. From past experience, we knew that the outpatient department register records the abovementioned domains with reasonable accuracy, and hence we assumed that it would present a robust sampling frame to ascertain the eligibility of participants with reference to maternal and/or child illness. Patients who were discharged after taking treatment were interviewed within the facility. Prior to interview, written consent was obtained from the patient and the interview was conducted separately in a congenial atmosphere. For patients who declined to be interviewed, the next patient from the register was selected to complete the required sample size. The study identified eligible respondents from out-patient records (and not in-patients) in order to minimise possible information bias emanating from the possibility that in-patients may have more severe illnesses leading to higher out-of-pocket expenditure (OOPE).

### Sample size

For the exit survey, a sample size of 309 was calculated. The assumptions used for this calculation were based on the known prevalence of 15% of maternal morbidity among women during the maternal period [7]. No such morbidity estimates among newborns of Pakistan were available. The derived sample was then divided equally across the three selected facilities. Hence, 103 clients in each facility were interviewed during the exit survey.

### Community survey

#### Subject selection and sampling procedure

The catchment population of each selected hospital was divided into two strata. The first stratum comprised of population that lay within a 5 km radius to the facility, mostly urban. The second comprised of population living more than a 5 km radius of the facility, mostly rural. Within the catchment areas, lists of women who had experienced an obstetric emergency or whose neonates had a health problem (within 28 days postnatally) during the last 6 months were obtained from the lady health workers of the selected areas. A 6-month duration was taken keeping in view the low ratio of obstetric complications and the required sample. In each stratum, systematic sampling with a random start methodology was adopted. The interval between two consecutive households for systematic sampling was determined for each stratum. The first household was taken randomly from the list.

### Sample size

A sample size of 690 for the community survey was calculated on the assumptions of known prevalence of 36% of mothers and 34% of newborns likely to utilise public sector health facilities for maternal and newborn morbidity [8]. The sample was then distributed equally across the catchment populations of each of the three selected facilities. Hence, in each district, 230 women were interviewed.

### Data collection and analysis for facility exit and community surveys

For both the surveys, locally hired female interviewers administered pre-tested structured questionnaires to obtain demographic information about the woman (i.e. household head, age, her and her husband’s education status, occupation, and monthly household income) and accessibility to the health facility (i.e. travel distance, travel time, travel cost, and waiting time for consultation at the facility). Descriptive statistics were calculated separately for public and private sector users.

### Focus group discussions (FGDs)

Three FGDs were held in the catchment area of each selected facility, each consisting of 6–8 participants. The first FGD was conducted with male elders and community notables, the second with prominent females and women’s groups, and the third with married women who went to a public or private health facility for an emergency health problem for herself or her newborn baby. These women were identified through hired community resource persons and social mobilisers with the help of the local population. Women who participated either in the exit or the community survey were not asked to be part of FGDs. Women who were poor and considered vulnerable due to religious, ethnic, geographical, and political affiliations/backgrounds were purposefully selected. A total of nine FGDs were conducted.

For FGDs, the data was collected through a study-specific, pre-tested, semi-structured tool translated in the local language. Notes were also taken in the local language and recorded. Transcripts were prepared in English. Common themes were derived and used to develop matrices to summarise and analyse the opinions of the respondents.

### Ethical considerations

Permission and clearance from the Director General of Health Services Sindh and respective District Health Officers of concerned districts was acquired. All the respondents were informed about the purpose of the study and their written consents were taken prior to interviews and group discussions. Information collected was retained under lock and key, while computerised datasets were password protected. Analysis used unique identifiers rather than personal information.

## Results

### Community and exit surveys

#### Demographic and reproductive characteristics of respondents

There were higher proportions of educated women amongst the participants of the community survey than those of the facility exit survey (43% vs. 27%). The proportion of educated husbands was also higher in the community survey than the facility exit survey (71% vs. 60%), which may suggest that public health facility users were concentrated among poorer segments of the catchment population. The majority of the community survey participants (70%) reported seeking care at a private health facility as opposed to a public facility (30%).

The large majority of women in both the community and exit surveys sought care from C-EmONC facilities for maternal health problems (75% vs. 90%, respectively) as opposed to neonatal health problems (12% vs. 9%, respectively). Only a small proportion of the participants in the community and exit surveys (13% vs. 1%, respectively) reported seeking care for both maternal and neonatal problems.

#### Barriers to care utilisation

Geographic and economic access to C-EmONC facilities (Table 1).

**Table 1:** Access to comprehensive emergency obstetric and newborn care facilities

**Categories**	**Household survey**			**Facility exit survey**		
	**Total (n = 690)**			**Total (n = 309)**		
	**Total**	**Within 5 km from hospital**	**More than 5 km from hospital**	**Total**	**Within 5 km from hospital**	**More than 5 km from hospital**
	**n (%)**	**n (%)**	**n (%)**	**n (%)**	**n (%)**	**n (%)**
Travel time to public facility, hours						
≤1/3	68 (38)	50 (54)	18 (21)	92 (30)	72 (48)	20 (13)
1/3–1	96 (53)	36 (39)	60 (68)	178 (58)	76 (51)	102 (64)
>1	16 (9)	6 (7)	10 (11)	39 (12)	2 (1)	37 (23)
Travel time to private facility, hours						
≤1/3	152 (36)	93 (56)	59 (23)			
1/3–1	232 (56)	69 (41)	163 (64)			
>1	36 (8)	5 (3)	31 (12)			
Out-of-pocket expense to travel to public health facility						
Yes	147 (82)	80 (87)	67 (76)	253 (82)	106 (71)	147 (93)
No	33 (18)	12 (13)	13 (24)	56 (18)	44 (29)	12 (7)
Amount of out-of-pocket expense to travel to public health facility, PKR						
<100	31 (22)	26 (33)	5 (8)	140 (55)	74 (70)	66 (45)
100–500	54 (37)	38 (48)	16 (24)	101 (40)	32 (30)	69 (47)
>500	62 (42)	16 (19)	46 (68)	12 (5)	0 (0)	12 (8)
Out-of-pocket expense to travel to private health facility						
Yes	403 (96)	163 (98)	240 (95)			
No	17 (4)	4 (2)	13 (5)			
Amount of out-of-pocket expense to travel to private health facility, PKR						
<100	1 (1)	0 (0)	1 (1)			
100–500	12 (3)	4 (3)	8 (3)			
>500	390 (97)	159 (97)	231 (96)			
Were travel costs to public facility affordable?						
Yes	66 (45)	41 (51)	25 (37)	112 (44)	61 (58)	51 (35)
No	81 (55)	39 (49)	42 (63)	141 (56)	45 (42)	96 (65)
How did you manage travel costs (public sector user)?						
Sold asset	21 (14)	11 (14)	10 (15)	2 (1)	1 (2)	1 (2)
Took loan	51 (35)	26 (33)	25 (37)	59 (42)	16 (36)	43 (45)
Charity from relatives	16 (11)	7 (9)	9 (13)	43 (30)	17 (38)	26 (27)
Other	59 (40)	36 (44)	23 (35)	37 (26)	11 (24)	26 (27)
Were travel costs to private facility affordable?						
Yes	121 (29)	45 (27)	76 (30)			
No	299 (71)	122 (73)	177 (70)			
How did you manage travel costs (private sector user)						
Sold asset	9 (3)	5 (4)	4 (2)			
Took loan	190 (64)	62 (51)	128 (72)			
Charity from relatives	2 (1)	2 (2)	0 (0)			
Other	98 (33)	53 (43)	45 (26)			

About half of the community survey respondents living within a 5 km radius reached the private or public hospital within 20 minutes, while the majority of their counterparts living more than 5 km took at least 20 minutes to reach the public or private hospital (79% and 76%, respectively). Results from the facility survey showed trends similar to the public facility users in the community survey.

Overall, 82% and 96% of those who availed themselves of a public and private C-EmONC facility, respectively, incurred OOPE to travel there. Those who were living more than 5 km from the facility incurred higher expenditure than those who resided in the 5 km radius. The majority of public sector facility users (55%) and private sector facility users (71%) considered the travel costs to be unaffordable. The proportion was higher for public sector facility users who live more than 5 km from the facility (63%) than those living within 5 km of the facility (49%). More than one third (35%) of public sector users and about two thirds (64%) of private sector users who could not afford travel expenses took loans, while those who sold assets were 14% and 3%, respectively. A higher proportion of respondents who took loans to cover the expenses were living more than 5 km from the health facility (among both public and private sector users). The costs of services was markedly higher among those who utilised private sector facilities (mean, PKR 12,047; SD, 953.8) compared to the public sector (mean, PKR 7,276; SD, 793.9). The finding, however, needs to be interpreted with care since the amount of OOPE for treatment is dependent on the type and severity of illness, for which information was not collected during the study.

Waiting time in health facilities (Table 2).

**Table 2:** Reported waiting time at the health facility

**Time wait after reaching facility, min**		**Public total (n = 180)**		**Private total (n = 420)**	
		**n**	**%**	**n**	**%**
Women	Immediately (<15)	78	43	269	64
	15–30	40	22	71	17
	31–45	17	9	21	5
	46–60	13	7	21	5
	>60	32	18	38	9
		**Public total (n = 52)**		**Private total (n = 116)**	
		**n**	**%**	**n**	**%**
Newborn	Immediately (<15)	33	63	78	67
	15–30	8	15	15	13
	31–45	3	6	11	9
	46–60	3	6	4	3
	>60	5	10	8	7

More than half of women who reported using a private facility (64%) and less than half (43%) of women who reported using a public facility were seen immediately. The mean waiting time across the public and private sector was not significantly different (*P* <0.63). Conversely, nearly one fifth (18%) of women using the public sector and less than one tenth (9%) in the private sector had to wait for more than 1 hour for services.

### Focus group discussions (FGDs)

The FGDs revealed that access to a C-EmONC facility was hampered by poverty, religion, ethnicity, caste, the attitude of hospital staff, and the private practices of public sector healthcare providers. The findings of the FGDs are described below.

#### Barriers to care

The poor cannot afford OOPE.

Participants in Tharparkar district expressed that they had serious access problems since they could not afford the cost of travelling to a public sector C-EmONC facility. They had to take loans from richer people with heavy mark ups to bear the cost of travelling and treatment. One participant mentioned that, in an emergency, a pregnant lady could not afford travel costs due to poverty, and her parents arranged a traditional birth attendant who delivered her baby at home. The newborn was very weak and the mother was unable to feed the baby due to her illness.

In Ghotki district, FGD participants mentioned that the people from castes, such as Machi, Kori, Mangi, and Shura among Muslims, and Bagri, Bheel, Menghwar, Harigen, Maleech, and Baziger among Hindus, were so poor that they could not even afford the cost of travelling and treatment in case of emergencies. Participants further narrated that, because of their ethnic and religious backgrounds and economic status, they were not given preference in consultation and treatment even in emergency situations and were commonly discriminated against by hospital staff members.

Staff attitude and fear of discrimination as barriers to accessing a public sector C-EmONC facility.

In a FGD in Ghotki, participants said that, because of poor staff attitude and fear of discrimination in public sector hospitals, they were reluctant to go to facilities even in emergency situations and preferred to go to a private facility despite the fact that they had to spend more on travelling and treatment.

Both male and female FGD participants in Tharparkar district narrated that people from a few Hindu castes, such as Kolhi, Bheel, and Menghwar, the illiterate, and the poor, were discriminated against by healthcare providers.

In the FGD with mothers in Jamshoro district, they identified the Seyal caste as vulnerable because their women were not allowed to go to hospitals for treatment even in cases of emergency during pregnancy. The Seyal women strictly observed pardah, the social practice of not permitting women to go outside home alone and covering their faces and heads when going outside. In case of obstetric emergency, Seyal women would take treatment from traditional birth attendants at home, and were not permitted or able to access facility-based services.

The private practice of public sector healthcare providers as a cause of the secondary delay.

FGDs revealed that healthcare providers in public facilities were said to be doing more than one job simultaneously. Patients were diverted from public hospitals to a private hospital which was commonly owned by those working in the public sector. Moving from one facility to another in an emergency situation caused the secondary delay and increased risks to the life of a patient and the cost of travel.

In an FGD in Tharparkar district, one participant recounted his visit to a public sector facility when his wife had an emergency in her pregnancy. The senior obstetrician was said to be on leave but when they visited her private hospital she was available there. Participants were of the opinion that difficulties in accessing services were deliberately created by public sector staff to divert patients from public sector facilities to private facilities. Another participant in Ghotki district described an issue faced by his sister-in-law, who visited the public facility during an emergency. Since the gynaecologist was not available, she had no option other than to visit her private clinic located nearby. A participant in Jamshoro district narrated a case of a relative who had four normal deliveries. During her fifth pregnancy, she had an emergency and went to a public facility where she was told that her baby would be delivered through an operation and forced her to go to her private clinic where she was delivered normally.

## Discussion

Herein, we have highlighted inequities with and barriers to access to a C-EmONC facility. Through both the household and facility exit surveys, we have attempted to understand access from the point of view of a woman facing an obstetric or neonatal emergency.

### Summary of survey findings

It was found that OOPE travel and health facility costs were perceived to be high amongst survey participants. Participants reported a variety of means of bearing these costs, including selling assets or acquiring loans or charity funds. The waiting time for public and private sector users was found to be very similar. The results reflected that the majority preferred to go to a private sector C-EmONC facility for both maternal and newborn care. Similar findings have been mentioned in a situation analysis of Sindh, where the use of services from the public sector was found to be significantly less than the private sector [7].

### Summary of FGD findings

The reasons for greater utilisation of private sector facilities were explored in FGDs. Most of the participants in FGDs said that they preferred to go to a private sector facility due to 24-hour availability of female healthcare providers, the better quality of care, availability of services, and attitude of staff. Participants reported a number of factors through the FGD, which highlighted perceived and experienced barriers to seeking care at public sector hospitals. These included high OOPE, discriminatory staff attitudes towards the illiterate, poorer, socioculturally considered lower castes, and non-Muslims. The absence of female care providers at public sector facilities, and the tendency of public sector employees to divert patients to their private practices (so that they could charge fees for services), was shared by participants from all the three districts. The possible economic impact on OOPE for the diverted service users was not ascertained during this research. Similar reasons for barriers to access and utilisation of care have been documented through existing research in Pakistan [8–10].

The views expressed by the community can be summarised as institutional and sociocultural barriers to accessing a C-EmONC facility. The major institutional barrier was the lack of services due to staff shortages, and particularly of female staff, forcing women to seek emergency care elsewhere, mostly from private sector facilities. Thus, the majority of women attend the private sector, as corroborated by findings from the household survey. Although not directly investigated in this study, existing research suggests that poor service structure and work environments are linked to the practice of public sector healthcare providers diverting women to private facilities [9]. Furthermore, this does not only cause a secondary delay and an increase in the risk of morbidity and mortality, but has implications for travel costs. Other factors, such as poor staff attitude and discriminatory behaviour, seem to be key barriers to accessing a public sector C-EmONC facility. When a woman faces an obstetric emergency, she remains uncertain as to which facility to attend for treatment, either to a public or a private C-EmONC facility. If a poor woman chooses a public sector facility because of free or low cost services, she still has to overcome the travel costs. If she gets through the first hurdle of covering travel expenses she may have to turn back to a private facility when she does not find the required services at the public sector facility or is faced with absence of female healthcare providers to deal with obstetric emergencies in the conservative societies of rural Sindh.

Among the sociocultural factors, poverty was stressed as an important barrier to accessing a C-EmONC facility. Our study highlighted that a considerable segment of the population cannot access a health facility in an emergency situation because of the travel costs entailed; most of the respondents could not afford these and had to manage them through loans, charity, or the sale of assets. This situation is very alarming since C-EmONC services, both from the public and private sectors, may simply not be accessible and affordable for the vast majority of the poor and marginalised women in the targeted districts. The findings correspond with previous evidence in the country [11]. A study by the Sustainable Development Policy Institute found that 33% of the population is living below the poverty line in Sindh [12]; Tharparkar district has the highest proportion (47%) of people living below the poverty line, while in Jamshoro district, 39% were living below the poverty line. The study considered lack of access to a health facility as a proxy indicator of poverty. Thus, the unaffordability of high travel cost was understandable given this poverty status.

### Recommendations

#### We present our recommendations based on the study findings as follows.

Attitudes, practices, and sociocultural factors in the community could be addressed through social and behaviour change communication programmes in the community. We recommend that the Sindh government takes measures to initiate such programmes in the community as well as amongst the workforce of public sector hospitals as their implementation is expected to help improve attitudes towards patients.

We also recommend that the Sindh government launches quality improvement initiatives, such as clinical training, development of procedures, audits, and near miss case reviews, as such initiatives will help to improve the quality of care in public sector hospitals.

The issue of public sector staff working in the private sector requires certain policy and legislative measures. We recommend that the Sindh government formulates appropriate policies and laws to regulate this.

A community transport system is a possible solution to addressing high travel costs. Such systems have been devised and tested through some pilot projects in the country. The government of Sindh should consider such projects to improve access to a C-EmONC facility and reduce the secondary delay in access to EmONC services in rural parts of the province [10].

Since OOPE at health facilities were perceived to be high among the survey participants, we also recommend that the Government of Sindh increases their budget for medical supplies and care, and develops a policy to subsidize services for patients.

Nearly a fifth of patients in the public sector and less than a tenth of patients in the private sector had to wait for more than an hour to receive services. Given that these were the patients in need of immediate medical attention at C-EmONC facilities, such long waiting times need to be addressed to minimize complications and adverse health outcomes due to delays in service provision (tertiary delay). Since this study highlighted that the majority of people went to private facilities, we recommend that the government undertakes legislative measures to regulate and ensure continuous quality of care in private facilities. Moreover, policy should be devised for the private sector to subsidize patients who cannot afford care.

## Conclusion

C-EmONC services in both public and private hospitals may simply be not accessible and affordable for the vast majority of poor and marginalised women in targeted districts of Sindh province.

## Abbreviations

B-EmONC: Basic emergency obstetric and newborn care
C-EmONC: Comprehensive emergency obstetric and newborn care
FGD: Focus group discussion
OOPE: Out-of-pocket expenditure
PKR: Pakistani Rupee
